# Supplementary material for: Muscle strengthening in individuals with Amyotrophic Lateral Sclerosis: a systematic review with meta-analyses
Source: PLoS One. 2025 Apr 24;20(4):e0320788. doi: 10.1371/journal.pone.0320788 (PMC12021160; doi:10.1371/journal.pone.0320788)
Supplement: S1 File — (DOCX) [file pone.0320788.s001.docx]

**S1 File. Search strategy**

**Terms for ALS (participants)**

1 amyotrophic lateral sclerosis

2 als

3 motor neuron disease

4 motor neurone disease

5 motoneuron disease

6 motoneurone disease

7 motor neuron disorders

8 lou gehrig

9 lou gehrig disease

10 lou gehrig syndrome

11 charcot disease

**Terms for intervention**

12 muscle strength

13 muscle strengthening

14 muscle power

15 muscle force

16 muscle torque

17 muscle training

18 resistance training

19 resistance exercise

20 strength training

21 motor function

22 movement

23 motor recovery

24 motor functioning

25 exercise

26 muscle exercise

27 rehabilitation

28 physiotherapy

29 physical therapy

**Terms for type of studies**

30 trial

31 clinical trial

32 control* trial

33 control*clinical trial

34 random* control* trial

35 random* trial

36 random* clinical trial

37 random* sampl*

38 random*

39 experimental*

40 experimental* design*

41 experimental study

42 experimental research

43 placebo

44 groups
