# Supplementary material for: Muscle strengthening in individuals with Amyotrophic Lateral Sclerosis: a systematic review with meta-analyses
Source: PLoS One. 2025 Apr 24;20(4):e0320788. doi: 10.1371/journal.pone.0320788 (PMC12021160; doi:10.1371/journal.pone.0320788)
Supplement: S2 File — (DOCX) [file pone.0320788.s002.docx]

**S2 File. Data extraction form**

**1. General information**

| Study title |  |
| --- | --- |
| First author and year of study |  |
| Name of the data extractor |  |
| DOI of the study |  |
| Date of extraction |  |
| Contact (email) of the author of the study |  |
| Requested information that was not in the study? |  |
| Did the author reply? What information did he/she provide? |  |
| Will any additional unpublished data provided by the authors be included in the review? |  |

**2. Study methodology**

| Study objective |  |
| --- | --- |
| Study design (as reported in the study; if not reported, how the reviewer classifies) |  |
| Instruments used to evaluate outcomes |  |
| Primary study outcomes (all) |  |
| Secondary study outcomes (all) |  |
| Total duration of the study (consider start: 1st assessment; end: last assessment) |  |
| Inclusion and exclusion criteria |  |
| Number of arms or groups (including control groups); |  |
| Source of funding (Include conflict of interest information) |  |
| Consent form provided? (Yes/No/Uncertain) |  |
| Ethical approval opinion (Yes/No/Uncertain) |  |

**3. Study characteristics – Participants**

| Description of study participants (as informed in the study) |  |
| --- | --- |
| Sample size (total number of participants included) |  |
| Average age of all groups (standard deviation) |  |
| Gender |  |
| Location of study |  |
| Institution or center (i.e., university hospital, home care, clinic, etc.) |  |
| Diagnostic criteria |  |
| ALS presentation form (bulbar/spinal/other) |  |
| Other health problems |  |

**4. Study characteristics - Interventions**

| Item | Notes and explanations | Intervention | Control |
| --- | --- | --- | --- |
| 1. Intervention name |  |  |  |
| 2. What was done? Intervention details |  |  |  |
| 3. Who (professional) provided the intervention? |  |  |  |
| 4. Where was the intervention provided? |  |  |  |
| 5. How long and how often will the intervention last? |  |  |  |
| 6. Was the intervention modified or adapted during the study? |  |  |  |

| Study numbers | Number |
| --- | --- |
| Eligible for inclusion |  |
| Excluded |  |
| Refused to participate |  |
| Randomized to the intervention group |  |
| Randomized to the control group |  |
| Excluded after randomisation | Intervention Group (reasons)– |
|  | Control Group (reasons)– |
| Sample losses | Intervention Group (reasons)– |
|  | Control Group (reasons)– |
| Follow-up losses | Intervention Group (reasons)– |
|  | Control Group (reasons)– |
| Included in the analysis (for each group, for each outcome) | Outcome 1 –  Intervention – Control– |
|  | Outcome 2 -  Intervention - Control– |
|  | Outcome 3 –  Intervention - Control– |
|  | Outcome 4 –  Intervention - Control– |
|  | Outcome 5 – Intervention-  Control– |
|  | Outcome 6 – Intervention-  Control– |

**6. Study characteristics – Outcomes and comparator groups**

| Outcome | Method of assessing the outcome (how the outcome was assessed, including instrument(s)) | Times of outcome assessment (including frequency and duration of follow-up) |
| --- | --- | --- |
|  |  |  |
|  |  |  |

**7. Data and results**

All data is number (of patients), not percentage.

***Dichotomous outcomes***

| Outcome | Timing of outcome assessment (e.g. baseline, immediate reassessment, late reassessment/follow-up) | Intervention group | | Group control | | Notes |
| --- | --- | --- | --- | --- | --- | --- |
|  |  | Observed (n) | Total (N) | Observed (n) | Total (N) |  |
|  |  |  |  |  |  |  |
|  |  |  |  |  |  |  |

***Continuous outcomes***

| Outcome | Timing of outcome assessment (e.g. baseline, immediate reassessment, late reassessment/follow-up) | Intervention group | | | Group control | | | Notes |
| --- | --- | --- | --- | --- | --- | --- | --- | --- |
|  |  | *Mean | Standard deviation | N | *Mean | Standard deviation | N |  |
| Muscle strength | Baseline |  |  |  |  |  |  |  |
|  | After  treatment |  |  |  |  |  |  |  |
|  |  |  |  |  |  |  |  |  |
|  |  |  |  |  |  |  |  |  |
